# Protecting procedural care—cybersecurity considerations for robotic surgery

**DOI:** 10.1038/s41746-022-00693-8

**Published:** 2022-09-20

**Authors:** William J. Gordon, Naruhiko Ikoma, Heather Lyu, Gretchen Purcell Jackson, Adam Landman

**Affiliations:** 1 Department of Medicine, Brigham and Women’s Hospital, Boston, MA USA; 2 Mass General Brigham, Somerville, MA USA; 3 Department of Biomedical Informatics, Harvard Medical School, Boston, MA USA; 4 Department of Surgical Oncology, University of Texas MD Anderson Cancer Center, Houston, TX USA; 5 Department of Pediatric Surgery, Pediatrics, Biomedical Informatics, Vanderbilt University Medical Center, Nashville, TN USA; 6 Intuitive Surgical, Inc., Sunnyvale, CA USA; 7 Department of Emergency Medicine, Brigham and Women’s Hospital, Boston, MA USA

**Keywords:** Technology, Hardware and infrastructure

## Abstract

Cybersecurity is an increasingly important concern for reliable healthcare delivery and is particularly salient for robotic surgery. Surgical robots are complex systems with numerous points of vulnerability, and there have been real-world demonstrations of successful cyberattacks on surgical robots. There are several ways to improve the risk profile of robotic surgery, including recognizing system complexity, investing in regular software updates, following cybersecurity best-practices, and increasing transparency for all stakeholders. As robotic surgery continues to technologically advance, ensuring overall system safety from a cybersecurity perspective is paramount.

## Background

The digitization of care delivery has had a profound impact on nearly all facets of healthcare. However, an increasing reliance on technology has created new risks, and healthcare has not been immune to the cybersecurity considerations plaguing other industries. Cybersecurity attacks have disabled entire hospital networks, delayed surgeries, diverted ambulances, and had significant operational disruptions worldwide^1–3^. Unfortunately, cybersecurity concerns are only increasing—70% of hospitals indicated having been victim to a recent significant security incident^4^.

Healthcare systems are uniquely vulnerable to cybersecurity attacks for several reasons. Health information is particularly valuable, creating financial incentive for attackers seeking patient data. Additionally, cybersecurity protection tends to be under-resourced by healthcare organizations, which are focused on care delivery and may have limited IT resources to spend on cybersecurity concerns. Finally, healthcare system attack surfaces are quite large. In addition to Electronic Health Records (EHRs), targets include hundreds and sometimes thousands of endpoints, such as patient devices (glucometers, pacemakers), hospital devices (infusion pumps, MRI scanners), medication dispensing systems, laboratory systems, anesthesia systems, to name only a few. Many devices are increasingly connected to the internet, creating further opportunities for attack. As an increasing public health concern^2^, cybersecurity will only become more relevant in the years to come.

One healthcare setting that is becoming more digital is procedural care. Through the procedural care spectrum—pre-, peri-, and post-operative—clinicians are increasingly relying on digital and technological capabilities to improve, augment, or enable procedures and operations. The international recognition of the importance of healthcare information security has accelerated over the past five years, driven by real-world events causing significant care disruptions, but much of the focus has been on system resiliency—ensuring that the information systems that operate healthcare organizations remain functional and online. The perceived risks are around downtime and disruption. However, the cybersecurity risks posed to perioperative care are uniquely concerning because of the active nature of surgery—operative interventions have an immediate, physical impact on patients, creating the potential for significant physical harm if attacked^5^. In this commentary, we explore the cybersecurity considerations for delivering procedural care, with a focus on robotic surgery as a driving use case.

## Robotic surgery: a use-case for cybersecurity considerations of procedural care

Robotic surgery is a good use-case for exploring the cybersecurity considerations of procedural care. Surgical robotics, which enable a surgeon to control robotic arms within a patient from a console, has had increased adoption over the past several decades. More than a third of general surgeons performed robotic surgery in 2021, up from 8.7% in 2018 and this number is growing across other surgical specialties, including gynecology, urology, otolaryngology, and cardiothoracic surgery^6–8^. Complex operations such as those requiring vascular dissection and reconstruction are increasingly being performed robotically, and hospitals are adapting to incorporate robotic surgery to all surgical practices and training. Modern surgical robots are complex, multi-layered systems, with unique mechanical and software controls that enable more precise tissue handling and motion scaling than other minimally invasive techniques^9^. Robotic surgery offers numerous advantages compared to non-robotic minimally invasive surgery, including better field visualization, dexterity benefits, operator ergonomics, and in some cases, improved clinical outcomes compared to other techniques^10^. In addition, although not being performed in routine practice, there is a technological capability to allow surgeons to remotely control robots to operate on patients residing in underserved areas, which is considered a potential solution to mitigate geographic disparity in access to surgical care. Disadvantages of robotic surgery include potentially increased costs, longer duration of procedures, and inconclusive clinical benefits depending on the surgical indication^10,11^.

## Cybersecurity risks of robotic surgery

The overall technical complexity of robotic surgery systems creates unique cybersecurity risks and harms. A breach of a surgical robot, like any tool with direct patient contact, could lead to immediate and critical physical harm. Cybersecurity concerns about other devices, such as implantable cardiac pacemakers have received widespread attention^12^. This risk is not an unwarranted futuristic fear. In 2015, Bonaci and colleagues demonstrated the technical ability to take over control of robotic function in a simulated environment, disrupting and overriding robotic functions^13^. More recently, several vulnerabilities were discovered in robots used to deliver medical supplies in a hospital^14^. Systems that can cause direct harm need physical *and* non-physical controls to reduce risk.

Contemporary surgical robots are multifaceted mechanical and digital platforms that integrate data and deliver insights using advanced computational methodologies to augment the procedural experience. Some robotic systems offer advanced imaging features to aid in identification of anatomic structures and evaluation of vascular perfusion; others provide telemonitoring and telepresence capabilities to facilitate training and intraoperative consultation. The integration of data sources, hardware, software, and networking necessary to accomplish these functions introduces new vulnerabilities and might enable an attack that could scale to multiple robots simultaneously.

Artificial intelligence techniques such as machine learning are finding numerous applications in the robotic surgery, such as identification of anatomic structures or operative tasks, prediction of procedure time, and improving visual tissue differentiation^15–17^. Artificial intelligence solutions often require significant data for development and a technical infrastructure for operation, and their performance can change over time, introducing additional cybersecurity risks. Beyond advanced computational methodologies, surgical robotics is also dependent on many communication, informational, and basic network technologies to operate. Intraoperative vendor communication, for example, is often helpful for troubleshooting, but is reliant on networked connections to sites outside of the hospital firewalls. Surgical data logs and patient videos are also points of attack, and breaches could compromise confidential preference data, surgeon-specific performance information, or patients’ personal health information. Robots are dependent on more general hospital infrastructure, like power, which could be interrupted. Robots require software updates and patches, which are points of vulnerability (e.g., through a man-in-the-middle or supply-chain attack, where the attacker may insert themselves between the robot and the server hosting the update software). Some robotic surgery platforms offer comprehensive subscription models that include cloud-based video recording hubs, performance tracking, and mobile access, all of which are further points of vulnerability. Finally, like other networked software devices, robotics could be compromised via physical means, become collateral damage from a wider attack, or fall victim to other traditional attacks, including deliberate employee misuse, denial of service attacks, or counterfeit equipment.

## Strategies to reduce cybersecurity risks for robotic surgery

Fortunately, there are numerous ways to mitigate and improve risk profiles for robotic surgery. First, organizations need to recognize the complexity of surgical robotics and build cybersecurity practices around this complexity. For example, surgical robots include hardware, firmware, and software; each will have different risks and strategies to improve safety. Hardware concerns, for example, may involve close relationships with manufacturers to reduce the risk that individual components like field-programmable gate arrays have not been compromised^18^. From a software perspective, device manufacturers need to invest heavily in building software with security in mind, and regularly updating software. The Da Vinci robot, for example, has regular software updates^19^. Other surgically adjacent features of the robot—like vendor support functionality, training capabilities, and video recordings—also need consideration and cybersecurity optimization. It should be carefully considered to what extent a robotics platform needs real-time internet connectivity during a procedure as a further way to lower risk—separate, private networks could also reduce risk.

Second, organizations should follow general, best-practice cybersecurity hygiene, including data encryption, anti-virus software, employee training, and a risk-based approach to cybersecurity^2,20^. Mitigation strategies, such as training OR staff on emergency robotic undocking^21^, threat identification, incident response planning are essential for preparedness. Individual employees remain the biggest organizational cybersecurity risk—it only takes one successful phishing campaign to elicit credentials that can give someone access to internal systems^22^. The US Food and Drug Administration (FDA) has published extensive pre-market and post-market documentation and guidance for device cybersecurity^23,24^, as has the European Medicine Agency, the US National Institute for Standards and Technology, the International Medical Device Regulators Forum, and others. Aligning organizational posture with best-practice guidelines will never guarantee cybersecurity safety, but will mitigate risk, and provide defensibility should there be an attack.

Finally, transparency for providers and patients involved in surgical robotic care is critical. While the expectation is that increased automation and reliance on data will improve value as well as clinical outcomes, the dependencies created by a highly networked and data-intensive surgical robot should be called out, and appropriate downtime procedures are needed. A surgery could be delayed if the robot is not functioning but addressing an intraoperative performance degradation or outage is far more complex. Identifying these hazards in a transparent fashion will enable a realistic assessment of the probability of occurrence, and lead to better downtime procedures. Vendors and providers must work together to understand what the risks are and focus on continuously reducing those risks as the technology continues to mature and advance. Further, in the event of a cybersecurity incident, vendors should have procedures for promptly notifying providers with complete information, enabling provider organizations to understand the risks and mitigate appropriately. Organizations such as the Health Information Sharing and Analysis Center (H-ISAC), HHS Health Sector Cybersecurity Coordination Center (HC3), or a Patient Safety Organization^25^ may help with communications.

## Conclusion

Robotic surgery is at the forefront of technology-driven care innovation. Like other areas of healthcare delivery that are increasingly dependent on technology, cybersecurity risk is an unfortunate reality. Cybersecurity concerns are particularly salient for robotic surgery because of the risk profile, which goes beyond merely downtime, and includes direct, physical patient harm. While these systems offer numerous advantages, there is an inherent vulnerability in any complex digital system. Understanding these risks while simultaneously working to reduce them will lead to safer and more reliable surgical care.

## Data Availability

Data sharing not applicable to this article as no datasets were generated or analyzed during the current study.
